# 2026 Bundibugyo virus outbreak: the role of genomics in Orthoebolavirus outbreaks

**DOI:** 10.1099/mgen.0.001754

**Published:** 2026-06-05

**Authors:** Charlotte J. Houldcroft

**Affiliations:** 1 Department of Genetics, University of Cambridge, Cambridge, CB2 3EH, UK

**Keywords:** genomic epidemiology, global health, outbreak, virology

## Abstract

Central Africa is currently experiencing its third recorded outbreak of Bundibugyo virus, one of three orthoebolaviruses capable of causing lethal haemorrhagic fevers in humans with person-to-person transmission. The first genomes from this outbreak have been made available, creating an opportunity to reflect on the role of genomics in previous ebolavirus outbreaks, the costs associated with genome sequencing and how they can be reduced, and future uses of genomic data for public health benefit.

In May 2026, the World Health Organization reported confirmed cases of Bundibugyo virus (BDBV) in the Democratic Republic of the Congo (DRC) and Uganda, leading to the declaration of a Public Health Emergency of International Concern on 17 May 2026 [1]. BDBV is one of three orthoebolaviruses, with Zaire (EBOV) and Sudan (SUDV) ebolaviruses, capable of causing fatal human disease with human-to-human transmission. Viral haemorrhagic fever outbreaks caused by orthoebolaviruses can have case fatality rates of 25–90%, depending on the species causing the disease [2]. By 18 May 2026, there were 8 confirmed cases of BDBV, with 246 suspected cases and 80 suspected deaths in the DRC. Two confirmed but unlinked cases, one fatal, were reported within 24 h in Kampala, Uganda. These individuals had travelled to Kampala from the DRC [1]. By 19 May 2026, this suspected case count had already doubled to 513, primarily in the DRC.

EBOV was first recognized in what is now the DRC and the DRC has experienced multiple zoonotic spillovers of EBOV over the last 30 years. However, BDBV was first described in Uganda in an outbreak in 2007 [2]. In this outbreak, BDBV had an estimated 25–40% fatality rate [2,4].

The three human ebolaviruses, EBOV, SUDV and BDBV, are >30% different from one another at the genomic level and have linear, non-segmented negative-sense RNA genomes. All have the characteristic *Orthoebolavirus* gene order *3′-NP-VP35-VP40-GP-VP30-VP24-L-5′* [5]. BDBV has a genome of 18,940 bp (RefSeq accession NC_014373.1 [2]), and as of 18 May 2026, there were 29 complete or near-complete patient-derived genomes in GenBank. An international team moved rapidly to generate the first BDBV genomes from clinical samples, making them available via Pathoplexus [6] and sharing initial analysis on the Virological platform [7]. Data from the 2012 outbreak of BDBV estimated a substitution rate of 0.84×10^−3^ subs site^−1^ year^−1^ [8], compared to estimates of ~1.2×10^−3^ subs site^−1^ year^−1^ for EBOV derived from multiple studies between 2013 and 2016 [9], suggesting BDBV could accumulate 1 to 2 SNPs per month.

Amidst the human tragedy of the 2014–2016 West African ebolavirus epidemic, one of the rare positive notes to emerge was the acceleration of testing and licensing of diagnostic tests, treatments and vaccines for the Zaire species of Ebola virus [orthoebolavirus zairense, (EBOV)] [10,13]. However, currently no vaccine or treatment is licensed for BDBV. There is some evidence from animal models that vaccines for EBOV may provide heterologous protection against BDBV [14,17], despite the ~30% genomic divergence [18] between the antigenic components of some of the vaccine candidates. Ervebo contains the glycoprotein gene from EBOV strain Kikwit [19], while the two-dose, two-vector Zabdeno/Mvabea combination vaccine contains the glycoproteins from EBOV strain Mayinga, SUDV strain Gulu and Marburg virus strain Musoke, and the nucleoprotein from Tai Forest virus [20].

The 2014 West African ebola epidemic also saw advances in the deployment of real-time, in-country genomics as an epidemiological tool to assist with epidemic management. In 2014, some of the earliest genome sequences were obtained with a range of methods, including PCR amplification, host rRNA depletion and mixed short- and long-read metagenomic and amplicon sequencing [21,23]. In the 2026 BDBV outbreak, initial genomes were generated with a target enrichment approach and sequenced on both short- and long-read platforms, reflecting the evolving use of genomic technologies to generate the first high-quality genomes on which much of the outbreak response may initially rely [7,24]. The estimated mutation rate of BDBV suggests it would be suitable for sequencing using overlapping PCR amplicon strategies employed for EBOV [23] during the 2014–2016 outbreak.

Deployment of real-time EBOV genomics provided powerful epidemiological insights into the origins and development of the 2014 outbreak, such as a divergence of the outbreak virus lineages from the closest known ancestor a decade before the outbreak was recognized, and genomic support for epidemiological inferences of sustained human-to-human transmission [22]. Genomics also helped identify cross-border transmission events [23]. The large pool of ebola survivors from the 2014 outbreak also led to the acknowledgement of viral persistence in immune-privileged tissues such as the eyes and testes, and of the risks that these cases can present for onwards transmission and reignition of outbreaks that had been thought to be controlled [25,27].

Sequencing of 11 cases from the 2012 DRC BDBV outbreak suggested that two separate spillover events occurred in the single recognized outbreak [8]. It is anticipated that genomics will play a role in this BDBV outbreak, as it did in 2014 for EBOV. Further lessons from SARS-CoV-2 may be applicable, such as using genomics to monitor escape from diagnostic tests [28], and the match between vaccines, therapeutics and key pathogen antigens [29,30].

Notwithstanding the knowledge that whole-genome sequencing can provide in outbreaks, there are challenges associated with implementation, including access to equipment, knowledge, reagents and funding. During the COVID-19 pandemic, it is estimated that half of the first 100,000 SARS-CoV-2 genomes sequenced in Africa were sequenced using Oxford Nanopore Technologies platforms [31]. Rapid sequencing of viral outbreaks is possible with a variety of technologies, including Illumina [32] and PacBio [33], but laboratory setup and maintenance costs can be high [34]. The use of portable sequencers and mobile laboratories is part of a broader shift towards sequencing happening in-continent, in-country and often on-site, in both higher and lower resource settings [35,36]. Samples no longer need to be shipped on dry ice to laboratories elsewhere in the world [22]. Work is ongoing to develop cheaper sequencing protocols, particularly for amplicon-based methods, that are compatible with a larger range of reagents when supply chains are under pressure [37]. Using Burkina Faso as an example, these cost-cutting protocols are estimated to reduce the price of a single library preparation from ~£52 sample^−1^ to £17 sample^−1^, for example by substituting a Moloney Murine Leukemia Virus (M-MLV) reverse transcriptase (RT) instead of a brand-name RT enzyme [37]. With reagents available at UK list prices, the total costs of sequencing a SARS-CoV-2 genome can be halved [37] compared to the costs of using a high-throughput SARS-CoV-2 sequencing protocol that was used in the UK (LoCost [38]).

In higher-income countries, genomic surveillance and outbreak-response sequencing are estimated to provide significant returns on investment through averted transmission events [39]. Continental pathogen surveillance efforts are recognized as a cost-effective alternative to establishing and maintaining permanent pathogen sequencing capacity in low-resource settings [34]. Many African nations have strong pathogen genomics infrastructure after decades of battling tuberculosis and HIV [40]. Recent multi-country outbreaks of mpox, as well as the SARS-CoV-2 pandemic [41,43], have led to further increases in the genomics capacities and knowledge bases in central African countries [44] and across the continent as a whole.

Local public health leadership must set epidemiological priorities for sequencing and diagnostics in the current outbreak [45]. It is likely, however, that international support, including funding [41], will be required to support the best use of BDBV for public health benefit; particularly as resources in the DRC are already stretched by existing challenges such as cholera, measles and plague [46]. In 2025, there were an estimated 48,000 cases of cholera, 17,000 cases of mpox, 36,000 cases of measles and 35 confirmed cases of Ebola virus disease. The estimated case fatality rates of these pathogens vary widely, from 47% for Ebola virus disease to 3% for cholera and 1.6% for measles [47]. Unlike BDBV, these other pathogens have effective vaccines and/or treatments, but access is poor for those in greatest need [47]. The same will be true for BDBV.

It is to be hoped that, in a world with an increasing number of zoonotic disease outbreaks, and attention drawn to emerging pathogens such as Hantavirus, international and academic solidarity can support the deployment of genomics in the BDBV virus outbreak to best support local public health priorities.

## Abbreviations

BDBV: Bundibugyo virus
DRC: Democratic Republic of the Congo
EBOV: Orthoebolavirus zairense
M-MLV: Moloney Murine Leukemia Virus
RT: reverse transcriptase
SUDV: Sudan ebolavirus
